# *WDR13*: A Novel Gene Implicated in Non-Syndromic Intellectual Disability

**DOI:** 10.3390/genes12121911

**Published:** 2021-11-28

**Authors:** Sylwia Rzońca-Niewczas, Jolanta Wierzba, Ewa Kaczorowska, Milena Poryszewska, Joanna Kosińska, Piotr Stawiński, Rafał Płoski, Jerzy Bal

**Affiliations:** 1 Department of Medical Genetics, Institute of Mother and Child, 01-211 Warsaw, Poland; milena.poryszewska@imid.med.pl (M.P.); jerzy.bal@imid.med.pl (J.B.); 2 Department of Internal and Pediatric Nursing, Faculty of Health Sciences with Institute of Maritime and Tropical Medicine, Medical University of Gdansk, 80-210 Gdansk, Poland; jolanta.wierzba@gumed.edu.pl; 3 Department of Biology and Medical Genetics, Medical University of Gdansk, 80-211 Gdansk, Poland; ekaczorowska@gumed.edu.pl; 4 Department of Medical Genetics, Warsaw Medical University, 02-106 Warsaw, Poland; joanna.kosinska@wum.edu.pl (J.K.); stawinski84@gmail.com (P.S.); rploski@wp.pl (R.P.)

**Keywords:** X-linked intellectual disability, X-chromosome exome sequencing, WD-40 protein family

## Abstract

Investigating novel genetic variants involved in intellectual disability (ID) development is essential. X-linked intellectual disability (XLID) accounts for over 10% of all cases of ID in males. XLID genes are involved in many cellular pathways and processes. Some of them are not specific to the development and functioning of the neural system. The implementation of exome sequencing simplifies the search for novel variants, especially those less expected. Here, we describe a nonsense variant of the XLID gene, *WDR13*. The mutation c.757C>T (p.Arg253Ter) was uncovered by X-chromosome exome sequencing in males with a familial form of intellectual disability. Quantitative PCR (qPCR) analysis showed that variant c.757C>T caused a significant decrease in *WDR13* expression in the patient's fibroblast. Moreover, it dysregulated other genes linked to intellectual disability, such as *FMR1*, *SYN1*, *CAMK2A*, and *THOC2*. The obtained results indicate the pathogenic nature of the detected variant and suggest that the *WDR13* gene interacts with other genes essential for the functioning of the nervous system, especially the synaptic plasticity process.

## 1. Introduction

Intellectual disability (ID) is a heterogeneous group of rare diseases characterized by deficits in intellectual and adaptive functioning of varying severity. X-linked forms of ID (XLID) account for over 10% of all male cases, including the most common disorder—the fragile X syndrome. The number of new genes implicated in XLID has increased rapidly over the last few years using next-generation sequencing (NGS). To date, more than 150 known genes have been reported.

The *WDR13* gene has been located on the p arm of the X-chromosome (Xp11.23). Singh et al. showed that *WDR13* encodes WD (tryptophan-aspartate) repeat-containing protein. This molecule is highly conserved and expressed in most tissues, with relatively higher expression observed in the brain, pancreas, ovaries, and testes [1]. The role of WDR13 in humans is not well understood, but the nuclear localization in the cell may suggest a regulatory function. More information is known about animal homologs, the WDR13 gene, and protein. *Wdr13* transcript was enriched following synaptogenic lesion of the hippocampus in rats. This finding suggests the neuroprotective role of the molecule [2].

Moreover, D’Agata and his group indicate that Wdr13 may be implicated in learning and memory abilities [3]. Additional studies have shown that the absence of Wdr13 protein predisposes mice to a depression-like phenotype with mild social isolation, anxiety, and chronic stress [4]. Gene expression analysis revealed that knock-out of the *Wdr13* gene leads to differential expressions of synaptic proteins, predominantly two of them: *Syn1* and *Camk2a*. The human homologs of both genes are involved in neural development and functioning. The *SYN1* gene is important for proper axono- and synaptogenesis. Pathogenic *SYN1* variants cause intellectual disability (OMIM#300115) or epilepsy with variable learning disabilities and behavior disorders (OMIM#313440). The *CAMK2A* gene is required for hippocampal long-term potentiation (LTP) and spatial learning. Pathogenic variants of this gene are a well-known cause of both recessive and dominant forms of intellectual disability (OMIM#:618095, 617798).

Little is known about the role of the WDR13 protein in the development of intellectual disability. The literature describes three variants that are likely causes of ID. Whibley et al. revealed intragenic deletion of the *WDR13* gene in a family with suspected XLID [5]. Another group found microduplication encompassing the *WDR13* in an 11-year-old boy with hyperactivity, learning, and visual–spatial difficulties [6]. The missense mutation (c.86C>T, p.Ala29Val) was identified in a male with an intellectual disability [7]. All reported variants are likely pathogenic according to in silico analysis results, but there is no evidence of the brain-specific function of the WDR13 in these patients.

Our study found a loss-of-function variant of the *WDR13* gene in a male patient with an intellectual disability whose family history indicates X-linked inheritance. Additional analyses showed that the lack of the *WDR13* gene in the patient's fibroblast caused dysregulation of the expression of several neural genes involved in the development of ID, including *FMR1*.

## 2. Materials and Methods

### 2.1. Informed Consent

The study was conducted according to the principles of the Declaration of Helsinki. Written informed consent was obtained from the patients. The Bioethics Committee of the Institute of Mother and Child, Warsaw, Poland, approved the study protocol (number 30/2012).

### 2.2. Clinical Description

The patient is a 22-year-old man with severe intellectual disability born to young, unrelated healthy parents at 33 weeks of a second pregnancy. A small amount of fetal water triggered premature birth. The patient's birth weight was 2400 g (5c<), height was 49 cm (25c–50c), and head circumference was 33 cm (5c<). His Apgar score was 10. He had an uneventful neonatal course and did not require an incubator. He was able to sit at 8 months and walk at about 18 months. His gait is ataxic. At the age of 2, he said his first word. His development was stimulated from the age of 4. In 22 years, he has demonstrated the level of development of a 7-year-old child. He can speak a complete sentence, read simple books, and copy letters and words. He has behavioral problems—aggression and stereotypies. The patient has been drooling since childhood. He has proper eyesight and hearing. As a child, he was suspected of having diabetes, but this was ruled out. He is slim but he does not demonstrate dysmorphic features.

**Figure 1:**
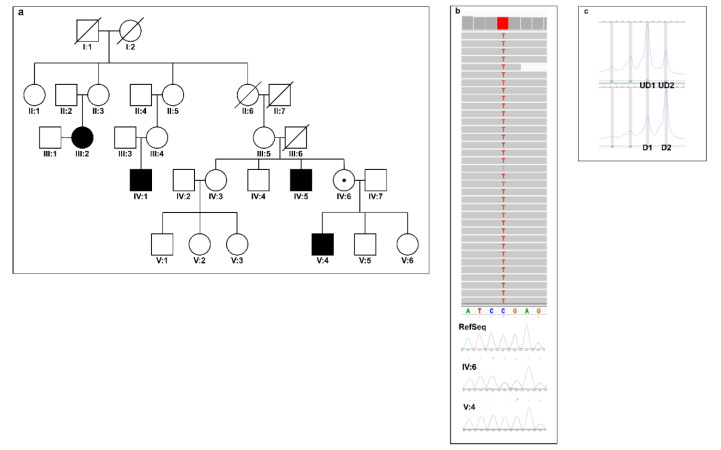
(**a**) Pedigree of the family; (**b**) Visualization of the X-chromosome exome sequencing results in the Integrative Genomics Viewer (IGV). Below are the Sanger sequencing results. The sequencing confirmed the hemizygous variant c.757C>T in the proband (IV:2) and heterozygous variant in the mother (III:2); (**c**) The result of the chromosome skewing is calculated as (D1/UD1)/(D1/UD1 + D2/UD2) when D1 and D2 represent the value of an area under the digested first, second peaks; UD1 and UD2 correspond to the area under undigested peak one and two.

He has a healthy older brother and two younger sisters. In his family, intellectual disability affects the mother's male relatives (Figure 1a). Patient III:4 has a moderate intellectual disability. He started speaking at the age of 4, but he uses incomplete sentences. He moves properly. Relative II:11 had an intellectual disability. No more clinical data about him are available.

### 2.3. Material Used in Genetic and Molecular Analysis

Peripheral blood was obtained from proband and his mother. Primary dermal fibroblasts from the patient and a healthy 20-year-old male were derived from a skin biopsy and cultured in Advance DMEM (Thermo Fisher Scientific Waltham, MA, USA) supplemented with 10% fetal bovine serum (Thermo Fisher Scientific, Waltham, MA, USA), 1x Glutamax (Thermo Fisher Scientific, Waltham, MA, USA), penicillin-streptomycin, and 2 mM L -glutamine (Merck, Darmstadt, Germany) in a 37 °C incubator with 5.0% CO_2_.

Genomic DNA was isolated from both blood and fibroblasts with Sherlock AX kit (A&A Biotechnology, Gdansk, Poland). Total RNA was extracted from fibroblasts using TRIzol Reagent (Thermo Fisher Scientific, Waltham, MA, USA). Protein lysate was isolated with RIPA buffer supplemented with a protease inhibitor cocktail (Thermo Fisher Scientific, Waltham, MA, USA).

### 2.4. The X-Chromosome Exome and Sanger Sequencing

The X-chromosome exome was sequenced using Agilent SureSelect Human X Chromosome Kit (Agilent) according to the manufacturer’s protocol. Genomic DNA (200 ng) was broken down into 150 bp fragments using COVARIS M220 (Covaris). Library preparation by applying the Agilent SureSelect system allowed for the analysis of 7591 exons of 745 genes of the human X chromosome (47 657 RNA baits). Paired-end deep sequencing was performed on the HiSeq1500 platform.

### 2.5. Quantitative Real-Time PCR (qRT-PCR)

Single-stranded cDNA was synthesized using the TranScriba Kit (A&A Biotechnology, Gdansk, Poland). Relative quantitation was performed for *WDR13*, *THOC2*, *NCBP1, FMR1*, *CAMK2A,* and *SYN1* genes. The duplex qRT-PCR reaction was conducted with an ABI7300 Genetic Analyzer (Applied Biosystems) using the FAM-labeled target TaqMan Probes (Thermo Fisher Scientific, Waltham, MA, USA, Table 1) and VIC-labeled reference TaqMan Probes (Table 1). Gene expression values were normalized to the expression of the reference genes. Normalized expression of genes in unaffected control was set to 1 and the ΔCt was calculated to determine the fold change of genes in affected patients. Expression data reflect the means of three independent experiments, each performed in triplicate.

### 2.6. X-Chromosome Inactivation

We analyzed the status of the X-chromosome inactivation for the proband mother. The analysis examines the highly polymorphic trinucleotide (CAG) repeats in the first exon of the human androgen-receptor gene (*AR*). Genomic DNA was digested with the methylation-sensitive restriction enzyme *HpaII* and amplified with primers according to the protocol described by Allen et al. [8]. The fragment size was analyzed using capillary electrophoresis. The cutoff value for skewed X-chromosome inactivation was set as 80, according to the report of Shvetsova et al. [9].

## 3. Results

### 3.1. The X-Chromosome Sequencing Reveals a Hemizygous Mutation in the WDR13 Gene

Subsequent X-chromosome sequencing and follow-up Sanger sequencing of genomic DNA from the proband identified hemizygous mutation NC_000023.11:g.48600552C>T (NM_001347217.2):c.757C>T (p.Arg253Ter) in the *WDR13* gene. The proband's mother is a heterozygous carrier of the variant (Figure 1b,c). Analysis of X-chromosome inactivation revealed skewing inactivation (81:19) in mother’s blood cells (Figure 1c). The mutation is not found in the general population (data based on Exome Aggregation Consortium Browser, Genome Aggregation Database, and 1000 Genomes).

The combined annotation dependent depletion (CADD) Phred scores, which rank the deleteriousness of single nucleotide variants within the human genome, were 36. The identified variant is predicted to be disease-causing by Mutation Taster (see URLs) [10], damaging by FATHMM-MKL, and deleterious by LRT. Webserver VarSome (see URLs) [11,12], which classifies genetic variants according to the ACMG/AMP 2015 guidelines [13], ranked the variant as pathogenic with the evidence codes PVS1, PM2, and PP3.

### 3.2. Gene Expression Analysis of WDR13 Mutations

**Figure 2:**
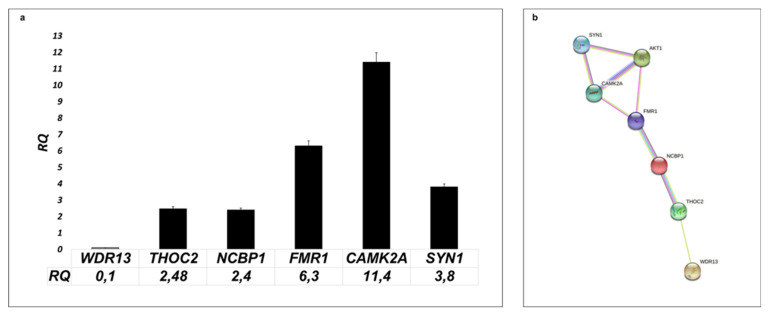
(**a**) Analysis of expression of selected synaptic genes in patient's fibroblast. RQ presented fold change of genes in affected patients normalized to the expression of genes in unaffected control (set to 1). (**b**) String analysis of protein encoding by analyzing patients' cells genes. Strings indicate both functional and physical protein associations and gene co-expression (https://string-db.org/, accessed on 20 October 2021).

The variant of *WDR13*, a conserved nonsense mutation in exon 5 of 9 (c.757C>T), is predicted to cause the premature stop of protein synthesis. We found that *WDR13* mRNA expression is lower in patients’ fibroblast compared to control (Figure 2a). The additional analysis showed upregulation of the expression of five genes involved in the development of intellectual disability (Table 1). The *CAMC2A* gene is 11.4 times more abundant in patients than in controls. The *FMR1* gene is expressed 6 times higher. The expression of the genes *SYN1, NCBP1*, and *THOC2* is 3.8, 2.4, and 2.48 times higher than control.

## 4. Discussion

WD40 is one of the most abundant and interactive domains in the eukaryotic genome. Proteins containing this domain are involved in many cellular pathways, such as cycle progression, signal transduction, apoptosis, and gene regulation. Their dysfunction contributes to the development of many diseases, including neurodevelopmental disorders [14,15].

We identified a novel nonsense variant (c.757C>T/p.Arg253Ter) in one of the WD40 members, the *WDR13* gene (Figure 2a). The mutation was found in the male with a severe intellectual disability, gait disturbance, aggression, and stereotypical movements through an X-chromosome sequencing approach. *WDR13* is a poorly characterized gene previously proposed as a candidate gene for X-linked intellectual disability [6,7,8]. The pathogenic mutations of its paralogue—*WDR26*—lead to well-recognized intellectual disability (Scraben-Deardorff syndrome, OMIM#617616) [16], demonstrating an overlap with the phenotype of our patient.

The identified mutation is a single nucleotide substitution, which probably causes nonsense-mediated decay (NMD) of the transcript due to premature termination codon activation (p.Arg253Ter). Gene expression analysis showed a significant decrease in the *WDR13* gene in patient fibroblasts, which may support this hypothesis (Figure 2a). Mitra et al. investigated the role of *Wdr13* in the brain on knock-out mice and revealed that the absence of the gene caused mild anxiety. Moreover, this research group showed that the lack of *Wdr13* led to the up-regulated expression of multiple genes, including synaptic genes such as *Syn1, Rab3a, Nrxn2,* and Camk2a. Surprisingly, Wdr13 null mice demonstrate better results in the Morris water maze task of spatial memory than wild-type animals [17]. Another study indicates that a lack of WDR13 protein impairs memory and learning efficiency [18]. There are no studies on human cells in the literature. Our analysis of the patient's fibroblasts is the first attempt to understand the molecular changes caused by the loss-of-function mutation of the *WDR13* gene. Concerning the results of WDR13 knock-out mice experiments, we investigated whether the lack of the *WDR13* gene in the patient's cells also influenced the expression of genes related to the functioning of synapses such as *CAMK2A*, *FMR1*, *THOC2,* and *SYN1*. Quantitative analysis showed increased expression of all examined genes with the most significant changes for known intellectual disability genes *CAMK2A* and *FMR1* (Figure 1a). The *CAMK2A* gene encodes Ca(2+)/calmodulin-dependent protein kinase engaged in synaptic plasticity. In the literature, both LoF and GoF variants of this gene are described as a cause of ID [19]. The *FMR1* gene, FMRP, is an RNA-binding protein that regulates the expression of critical genes in neural development, neuronal function, and synaptic plasticity [20,21]. The mutation of this gene leads to the most frequent monogenic form of intellectual disability, the fragile X syndrome [22]. The lack of both genes disturbs crucial synaptic plasticity processes, long-term potentiation (LTP), and long-term depression (LTD) [23].

Moreover, CAMK2A and FMRP proteins play a pro-survival role [24]. Jeon, et al., showed that overexpression of FMRP alleviated cell death, increased Akt activity, and enhanced Bcl-xL production [25]. Studies in pancreatic cells have shown that the lack of the WDR13 protein activates the PI3K/Akt pathway [26]. With this in consideration, we hypothesize that a lack of WDR13 causes the over-activation of the PI3K/Akt cell signaling pathway, which results in increased expression of CAMK2A and FMRP. String protein analysis shows experimentally that these two proteins interact (Figure 2b). Moreover, we observed increased expression of *SYN1* and*THOC2* genes in patients' cells (Figure 2a). Synaptin 1 is a neuronal phosphoprotein that regulates axonogenesis and synaptogenesis. The protein serves as a substrate for several different protein kinases, including AKT. Mutations in this gene are associated with X-linked intellectual disability (OMIM#300115) [27]. THOC2 (THO complex subunit 2) is part of the TREX complex engaged in transcription, processing, and nuclear export transport of mRNA. Variants of this gene are known to cause non-specific X-linked ID (OMIM #300957) [28].

In our research, we also found increased expression of the gene NCBP1 (Figure 2a). This nuclear cap-binding protein 1 (NCBP1) is necessary for capped RNA processing and intracellular localization. It has been reported that the silencing of NCBP1 results in cell growth reduction. Moreover, this protein interacts with FMRP and THOC2, which links the genes under study. Nothing is known about the role of this gene in ID [29].

Up-regulation of all described genes in the patient’s fibroblast proves that the identified variant c.757C>T of the *WDR13* gene is pathogenic and, probably through PI3K/Akt signaling pathway activation, causes changes in the functioning of synapses. Dysregulation of synaptic plasticity implicates cognitive and memory impairments associated with many neurological diseases, including intellectual disability. All overexpressed genes are involved in this process. We hypothesized that lack of WDR13 through NCBP1 caused changes in the expression *FMR1* gene. Additional changes are a consequence of the upregulation of *FMR1,* a gene essential for the proper functioning of the neural system. This scenario seems likely but requires additional study.

This is the first report indicating the involvement of a nonsense *WDR13* variant in the pathogenesis of non-syndromic ID. Therefore, it is necessary to conduct detailed research that would allow for a better understanding of the process of intellectual disability development related to the *WDR13* gene.

## 5. Conclusions

In conclusion, we present a comprehensive description of the patient with a pathogenic variant in the novel XLID gene-WDR13. The mutation is the cause of non-syndromic intellectual disability in the proband and his family. We establish that decreasing *WDR13* gene expression in patient fibroblast upregulates expression of other ID genes involved in the process of synaptic plasticities such as *FMR1*, *CAMK2A*, *SYN1*, and *THOC2*.

## Figures and Tables

**Table 1:** List of probes used for qPCR.

	Gene	RefSeq	Probe ID	Exon Boundary
Target	*WDR13*	NM_001166426.1	Hs01026404_g1	1–2
	*THOC2*	NM_001081550.1	Hs00396154_m1	33–34
	*NCBP1*	NM_002486.4	Hs00916644_m1	11–12
	*FMR1*	NM_001185075.1	Hs00924547_m1	15–16
	*CAMK2A*	NM_015981.3	Hs00392405_m1	15–16
	*SYN1*	NM_006950.3	Hs00199577_m1	5–6
Reference	*GAPDH*	NM_001256799.2	Hs03929097_g1	8–8
	*TBN*	NM_001172085.1	Hs00427620_m1	2–3

## Data Availability

The data that support the findings of this study are available from the corresponding author upon reasonable request.

## References

[1] Singh B.N., Suresh A., UmaPrasad G., Subramanian S., Sultana M., Goel S., Kumar S., Singh L. (2003). A highly conserved human gene encoding a novel member of WD-repeat family of proteins (WDR13). Genomics.

[2] Suresh A., Shah V., Rani D.S., Singh B.N., Prasad G.U., Subramanian S., Kumar S., Singh L. (2005). A mouse gene encoding a novel member of the WD family of proteins is highly conserved and predominantly expressed in the testis (Wdr13). Mol. Reprod. Dev..

[3] D’Agata V., Schreurs B.G., Pascale A., Zohar O., Cavallaro S. (2003). Down regulation of cerebellar memory related gene-1 following classical conditioning. Genes Brain Behav..

[4] Mitra S., Sameer Kumar G.S., Tiwari V., Lakshmi B.J., Thakur S.S., Kumar S. (2016). Implication of Genetic Deletion of Wdr13 in Mice: Mild Anxiety, Better Performance in Spatial Memory Task, with Upregulation of Multiple Synaptic Proteins. Front. Mol. Neurosci..

[5] Whibley A.C., Plagnol V., Tarpey P.S., Abidi F., Fullston T., Choma M.K., Boucher C.A., Shepherd L., Willatt L., Parkin G. (2010). Fine-scale survey of X chromosome copy number variants and indels underlying intellectual disability. Am. J. Hum. Genet..

[6] El-Hattab A.W., Bournat J., Eng P.A., Wu J.B., Walker B.A., Stankiewicz P., Cheung S.W., Brown C.W. (2011). Microduplication of Xp11.23p11.3 with effects on cognition, behavior, and craniofacial development. Clin. Genet..

[7] Hu H., Kahrizi K., Musante L., Fattahi Z., Herwig R., Hosseini M., Oppitz C., Abedini S.S., Suckow V., Larti F. (2019). Genetics of intellectual disability in consanguineous families. Mol. Psychiatry.

[8] Allen R.C., Zoghbi H.Y., Moseley A.B., Rosenblatt H.M., Belmont J.W. (1992). Methylation of HpaII and HhaI sites near the polymorphic CAG repeat in the human androgen-receptor gene correlates with X chromosome inactivation. Am. J. Hum. Genet..

[9] Shvetsova E., Sofronova A., Monajemi R., Gagalova K., Draisma H., White S.J., Santen G., Chuva de Sousa Lopes S.M., Heijmans B.T., van Meurs J. (2019). BIOS consortium, & GoNL consortium. Skewed X-inactivation is common in the general female population. Eur. J. Hum. Genet..

[10] https://www.mutationtaster.org/MT69/MutationTaster69.cgi?position_be=757&transcript_stable_id_text=ENST00000218056&transcript_stable_id_radio=ENST00000218056&gene=WDR13&sequence_type=CDS&new_base=T.

[11] Kopanos C., Tsiolkas V., Kouris A., Chapple C.E., Albarca Aguilera M., Meyer R., Massouras A. (2019). VarSome: The human genomic variant search engine. Bioinformatics.

[12] https://varsome.com.

[13] Harrison S.M., Biesecker L.G., Rehm H.L. (2019). Overview of Specifications to the ACMG/AMP Variant Interpretation Guidelines. Curr. Protoc. Hum. Genet..

[14] Holt R.J., Young R.M., Crespo B., Ceroni F., Curry C.J., Bellacchio E., Bax D.A., Ciolfi A., Simon M., Fagerberg C.R. (2019). *De novo* Missense Variants in *FBXW11* Cause Diverse Developmental Phenotypes Including Brain, Eye, and Digit Anomalies. Am. J. Hum. Genet..

[15] Ben-Omran T., Fahiminiya S., Sorfazlian N., Almuriekhi M., Nawaz Z., Nadaf J., Khadija K.A., Zaineddin S., Kamel H., Majewski J. (2015). Nonsense mutation in the *WDR73* gene is associated with Galloway-Mowat syndrome. J. Med. Genet..

[16] Skraban C.M., Wells C.F., Markose P., Cho M.T., Nesbitt A.I., Au P.Y.B., Begtrup A., Bernat J.A., Bird L.M., Cao K. (2017). *WDR26* Haploinsufficiency Causes a Recognizable Syndrome of Intellectual Disability, Seizures, Abnormal Gait, and Distinctive Facial Features. Am. J. Hum. Genet..

[17] Mitra S., Sameer Kumar G.S., Jyothi Lakshmi B., Thakur S., Kumar S. (2018). Absence of *Wdr13* Gene Predisposes Mice to Mild Social Isolation—Chronic Stress, Leading to Depression-Like Phenotype Associated With Differential Expression of Synaptic Proteins. Front. Mol. Neurosci..

[18] Price M., Lang M.G., Frank A.T., Goetting-Minesky M.P., Patel S.P., Silviera M.L., Krady J.K., Milner R.J., Ewing A.G., Day J.R. (2003). Seven cDNAs enriched following hippocampal lesion: Possible roles in neuronal responses to injury. Brain Res. Mol. Brain Res..

[19] Küry S., van Woerden G.M., Besnard T., Proietti Onori M., Latypova X., Towne M.C., Cho M.T., Prescott T.E., Ploeg M.A., Sanders S. (2017). De Novo Mutations in Protein Kinase Genes *CAMK2A* and *CAMK2B* Cause Intellectual Disability. Am. J. Hum. Genet..

[20] Pfeiffer B.E., Huber K.M. (2009). The state of synapses in fragile X syndrome. Neuroscientist.

[21] Darnell J.C., Mostovetsky O., Darnell R.B. (2005). FMRP RNA targets: Identification and validation. Genes Brain Behav..

[22] Fu Y.H., Kuhl D.P., Pizzuti A., Pieretti M., Sutcliffe J.S., Richards S., Verkerk A.J., Holden J.J., Fenwick R.G.J., Warren S.T. (1991). Variation of the CGG repeat at the fragile X site results in genetic instability: Resolution of the Sherman paradox. Cell.

[23] Zeier Z., Kumar A., Bodhinathan K., Feller J.A., Foster T.C., Bloom D.C. (2009). Fragile X mental retardation protein replacement restores hippocampal synaptic function in a mouse model of fragile X syndrome. Gene Ther..

[24] Kool M.J., Proietti Onori M., Borgesius N.Z., van de Bree J.E., Elgersma-Hooisma M., Nio E., Bezstarosti K., Buitendijk G., Aghadavoud Jolfaei M., Demmers J. (2019). CAMK2-Dependent Signaling in Neurons Is Essential for Survival. J. Neurosci. Off. J. Soc. Neurosci..

[25] Jeon S.J., Han S.H., Yang S.I., Choi J.W., Kwon K.J., Park S.H., Kim H.Y., Cheong J.H., Ryu J.H., Ko K.H. (2012). Positive feedback regulation of Akt-FMRP pathway protects neurons from cell death. J. Neurochem..

[26] Fu Y., Li S., Tong H., Li S., Yan Y. (2019). WDR13 promotes the differentiation of bovine skeletal muscle-derived satellite cells by affecting PI3K/AKT signaling. Cell Biol. Int..

[27] Guarnieri F.C., Pozzi D., Raimondi A., Fesce R., Valente M.M., Delvecchio V.S., Van Esch H., Matteoli M., Benfenati F., D’Adamo P. (2017). A novel SYN1 missense mutation in non-syndromic X-linked intellectual disability affects synaptic vesicle life cycle, clustering and mobility. Hum. Mol. Genet..

[28] Kerr B., Gedeon A., Mulley J., Turner G. (1992). Localization of non-specific X-linked mental retardation genes. Am. J. Med. Genet..

[29] Mazza C., Ohno M., Segref A., Mattaj I.W., Cusack S. (2001). Crystal structure of the human nuclear cap binding complex. Mol. Cell.

